# Vein of Galen Aneurysmal Malformation: A Case Report With Insights Into Radiological Diagnosis Using Ultrasonography and Magnetic Resonance Imaging

**DOI:** 10.7759/cureus.100842

**Published:** 2026-01-05

**Authors:** Atul Kumar, Pragya Chaturvedi, Shrea Gulati, Sushma Pandey

**Affiliations:** 1 Radiodiagnosis, Medanta Hospital, Lucknow, IND

**Keywords:** anasarca, magnetic resonance imaging, role of radiology, tachypnoea, ultrasonography, vein of galen malformation

## Abstract

This report details the comprehensive radiological evaluation of a vein of Galen aneurysmal malformation in a neonate, using ultrasonography and magnetic resonance imaging. Vein of Galen malformation is a rare, congenital cerebral arteriovenous fistula and the leading cause of life-threatening high-output cardiac failure in neonates. A late preterm male infant presented with worsening tachypnea and generalized body swelling. Initial cranial ultrasonography revealed a large, midline anechoic structure with intense flow on color Doppler. Subsequent multiplanar magnetic resonance imaging and magnetic resonance angiography precisely delineated the neurovascular anatomy, showing a markedly dilated median prosencephalic vein, arterial supply from the left posterior cerebral artery, and the absence of normal deep venous drainage. This case highlights the critical, complementary roles of ultrasonography for rapid, accessible diagnosis and hemodynamic assessment, and magnetic resonance imaging for definitive anatomical mapping and pretreatment planning.

## Introduction

A vein of Galen aneurysmal malformation (VGAM) is a complex, non-hereditary congenital vascular anomaly characterized by abnormal arteriovenous shunts linking cerebral arteries directly to the median prosencephalic vein of Markowski. This embryonic structure is a precursor to the vein of Galen [1]. The pathological increase in blood flow through these connections results in aneurysmal dilation of the developing venous system, leading to significant clinical consequences. If not addressed, VGAM can lead to severe morbidity and historically documented high mortality rates, necessitating prompt and precise radiological evaluation for effective triage, prognostic assessment, and intervention planning [2]. The utilization of non-invasive imaging modalities, particularly ultrasound and magnetic resonance imaging (MRI), is crucial for the initial diagnosis and detailed anatomical delineation of the malformation. These advanced imaging techniques enable comprehensive visualization of the involved vascular structures, facilitating timely and informed intervention [3]. Recent advancements in treatment methods, particularly endovascular embolization techniques, have markedly transformed the management of vein of Galen malformation. This approach not only offers the potential for complete resolution of the malformation but also provides significant palliative care options for affected individuals. In this context, the role of radiology transcends conventional diagnosis; it serves as a critical roadmap for navigating the intricate and challenging treatment landscape associated with this condition. This case report delves into the imaging findings and the diagnostic trajectory employing these essential modalities.

## Case presentation

A late preterm male infant was born at 35 weeks of gestation via spontaneous normal vaginal delivery. The baby cried immediately after birth. Initially, the baby was well and was discharged on breastfeeding. On the seventh day of life, the baby developed yellowish discoloration of the entire body. The condition transiently improved after giving phototherapy. At one month of life, the patient presented with tachypnea and was diagnosed with respiratory distress. The baby was intubated in view of respiratory distress and was put on a mechanical ventilator.

Physical examination

The patient appeared sick on admission. Temperature was 34.5 degrees Celsius, suggestive of hypothermia. Heart rate was 90 bpm, suggestive of bradycardia. Capillary refill time was increased to >3 s. There was significant generalized edema noted.

Systemic examination

Neurological and cardiovascular examination was unremarkable. Respiratory examination revealed an increased respiratory rate (>60/min). Abdominal examination was unremarkable.

Pathological investigations

Blood counts were unremarkable. Erythrocyte sedimentation rate and C-reactive protein were markedly increased. Serum sodium and serum potassium levels were increased, and serum calcium levels were decreased. Blood culture was negative. Direct bilirubin, indirect bilirubin, and total bilirubin were increased. Renal function tests were unremarkable. Newborn screening panel for congenital errors of metabolism, immunodeficiencies, and endocrine diseases was done and was found to be normal. Ultrasound of the whole abdomen was done, which revealed edema along the gall bladder wall and gall bladder sludge. Ultrasound of the cranium was done, which revealed a complex arteriovenous malformation (vein of Galen). The 2D echo was unremarkable.

The clinical and laboratory findings indicated that the patient was in shock. Although heart failure was a primary concern, it was excluded following a 2D echocardiogram. The patient received symptomatic treatment, and an endovascular embolization procedure is planned once the patient achieves stabilization.

Imaging findings

Ultrasonography

A high-frequency phased-array transducer was used via the anterior fontanelle in coronal and sagittal planes. A comprehensive grayscale and color Doppler analysis was performed. Grayscale imaging demonstrated a large, well-defined, anechoic structure in the suprasellar cistern. Color Doppler revealed turbulent flow.

Magnetic Resonance Imaging

A large complex vascular malformation was seen in the quadrigeminal cistern, which was seen extending to the suprasellar region. It appeared as a flow void on both T1W and T2W sequences due to rapid blood flow.

**Figure 1:**
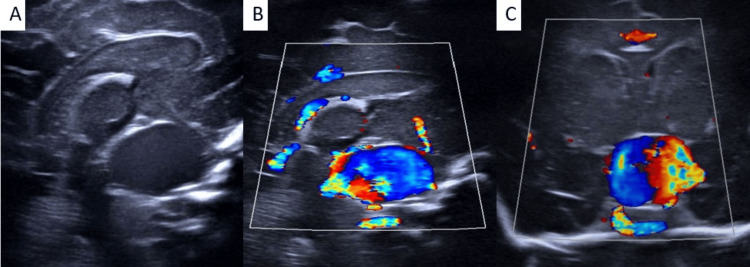
Transcranial ultrasound (A) Grayscale transcranial ultrasound (sagittal view) image shows a well-defined anechoic cystic structure at the suprasellar region. (B, C) On color Doppler transcranial ultrasound (sagittal and coronal views), the anechoic structure shows increased flow with marked aliasing suggestive of turbulent flow.

**Figure 2:**
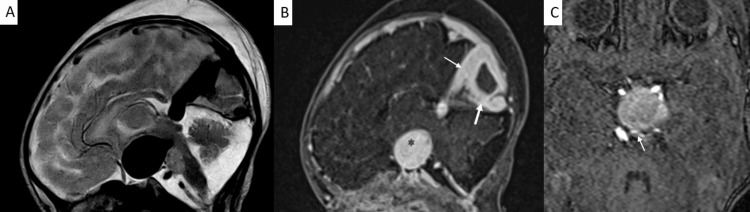
MRI brain with contrast (A) On T2 sagittal MRI images, there is a linear complex vascular malformation at the quadrigeminal cistern reaching anteriorly into the dilated median prosencephalic vein in the suprasellar region showing T2 flow void. (B) T1 post-contrast sequences reveal a complex vascular malformation showing marked contrast enhancement. The gray star represents a dilated median prosencephalic vein. The thin white arrow represents the falcine sinus, and the thick white arrow represents the straight sinus. (C) 3D time-of-flight images show the arterial supply. The white arrow shows the communication of the left posterior cerebral artery into the dilated median prosencephalic vein. MRI: magnetic resonance imaging

Both non-contrast time-of-flight magnetic resonance angiography and contrast-enhanced magnetic resonance demonstrated a delineated vascular architecture. It was supplied by arterial feeders arising from the left posterior cerebral artery, suggesting type I malformation (Yasargil classification). The dilated vein drained posteriorly via a persistent falcine sinus, a fetal venous channel that courses within the falx cerebri to reach the superior sagittal sinus. No associated parenchymal hemorrhage or ischemia was seen (Figures 1, 2).

## Discussion

The progression of imaging techniques from ultrasonography to MRI offers a comprehensive diagnostic overview. As the first and most readily available method, ultrasonography is crucial. In the appropriate clinical context, the discovery of a midline cystic structure with turbulent flow is characteristic of a specific condition [4]. Its advantages are found in the capability for real-time hemodynamic evaluation-the arterialized spectral waveform with a low resistive index within the venous structure indicates the presence of an arteriovenous fistula. Additionally, cranial ultrasonography can be conducted in a portable manner, which makes it essential for monitoring unstable neonates. However, its drawbacks include reliance on the operator’s skill, a restricted field of view for observing the feeders, and a lack of definitive characterization of the venous drainage pattern or the ability to rule out related anomalies [5].

MRI is the primary non-invasive tool for anatomical characterization due to its exceptional soft-tissue resolution and ability to capture images in multiple planes. It validates diagnoses, accurately illustrates blood vessels, and evaluates secondary effects such as mass effect, hydrocephalus, and parenchymal bleed [3,6]. In this case, MRI was crucial for classifying the arteriovenous malformation and identifying the falcine sinus as the dominant drainage pathway [7].

The imaging-based differential diagnosis includes alternative reasons for a midline cystic mass, such as an arachnoid cyst or a pineal cyst. The presence of high-flow vascular signals on Doppler imaging and flow voids in MRI effectively rules out non-vascular causes. The treatment for vein of Galen malformation has been transformed by endovascular embolization, which now provides the possibility of a cure or considerable palliative care [8]. The role of radiology extends beyond diagnosis to providing the essential roadmap for this treatment.

## Conclusions

This radiological case report discusses a systematic imaging approach for a neonate diagnosed with a vein of Galen malformation. Cranial ultrasound is an excellent, rapid screening tool that provides crucial anatomical information strongly indicative of the condition. Following this, multisequence MRI and magnetic resonance angiography are essential steps, offering a comprehensive and detailed anatomical roadmap of the malformation, along with insights into its vascular supply, drainage, and potential complications. The combined use of these non-invasive modalities enables a confident diagnosis and accurate classification and provides vital data for preoperative planning required for life-saving endovascular interventions.
